# COVID-19: Epidemiologische und klinische Fakten

**DOI:** 10.1007/s00117-020-00741-y

**Published:** 2020-08-31

**Authors:** Christoph J. Hemmer, Hilte F. Geerdes-Fenge, Emil C. Reisinger

**Affiliations:** grid.413108.f 0000 0000 9737 0454 Abt. f. Tropenmedizin und Infektionskrankheiten, Universitätsmedizin Rostock, Ernst-Heydemannstraße 6, 18055 Rostock, Deutschland

**Keywords:** SARS-CoV‑2, Polymerase-Kettenreaktion, Zytokinantwort, Akutes Lungenversagen, Impfung, SARS-CoV‑2, Polymerase chain reaction, Cytokine response, Acute respiratory distress syndrome, Vaccination

## Abstract

Bis zum 31.07.2020 wurden weltweit ca. 17,6 Mio. SARS-CoV-2-Infizierte und ca. 680.000 Todesfälle aufgrund von COVID-19 gemeldet. SARS-CoV‑2 wird über Tröpfchen und wahrscheinlich auch Aerosole übertragen. Die Infektiosität beginnt 2–3 Tage vor Symptombeginn, auch asymptomatisch Infizierte sind infektiös. Die Erkrankung betrifft die oberen Atemwege und die Lungen (Pneumonie, akutes Lungenversagen [ARDS]), ferner Herz, Leber, Magen-Darm-Trakt und andere Organe. Das Virus nutzt ACE2 als Rezeptor zum Eindringen in Wirtszellen. Vaskulitis, Endothelschaden, Thromboembolien und Organversagen werden von einer massiven Zytokinantwort begleitet. Gefährdet sind vor allem Ältere sowie Personen mit Vorerkrankungen. Eine effektive antivirale Therapie ist bisher nicht verfügbar. Schwer kranke Patienten profitieren wahrscheinlich von Dexamethason und von frühzeitiger Therapie der Komplikationen. Impfstoffkandidaten befinden sich derzeit in der klinischen Prüfung.

## Abstract (en)

Until July 31, 2020, about 17.6 million SARS-CoV‑2 infections and 680,000 deaths from COVID-19 were reported. SARS-CoV‑2 is most likely transmitted by droplets and probably by aerosols. Patients become infectious 2–3 days before the onset of symptoms, and persons with asymptomatic infections are also infectious. COVID-19 affects the upper respiratory tract, lungs (pneumonia, acute respiratory distress syndrome [ARDS]), heart, liver, gastrointestinal tract, and other organs. SARS-CoV‑2 uses ACE2 a receptor to enter host cells. Vasculitis, endothelial damage, thromboembolic events and organ failure are accompanied by a massive cytokine response. Elderly people and those with pre-existing diseases are particularly vulnerable. An efficient antiviral therapy is not yet available. Severely ill patients may benefit from dexamethasone and early treatment of complications. Candidate vaccines are currently being tested in clinical studies.

Im Dezember 2019 meldeten die chinesischen Behörden ein Cluster von 27 Pneumoniefällen unbekannter Ätiologie in der chinesischen Großstadt Wuhan (Provinz Hubei) an die Weltgesundheitsorganisation (WHO; [59]). Am 7. Januar 2020 gaben die chinesischen Gesundheitsbehörden die Gensequenz des Erregers, eines neuartigen Coronavirus, bekannt, das zunächst 2019-nCoV, dann SARS-CoV‑2 genannt wurde. Die Erkrankung erhielt den Namen „Coronavirus Disease 19“ (COVID-19).

Wenige Tage später, am 20. Januar 2020, nahm eine Geschäftsreisende aus Shanghai/China an einem Workshop in München teil. Erst nach ihrer Rückkehr nach China am 22.01.2020 wurde bei ihr und in den folgenden 4 Wochen bei 16 direkten und indirekten Kontaktpersonen in Deutschland COVID-19 festgestellt [4].

Ein zweiter Ausbruch in Deutschland betraf die Teilnehmer an einer Karnevalssitzung am 15.02.2020 im Kreis Heinsberg; dort wurden bis zum 22. April 2020 insgesamt 1739 Fälle bestätigt [25]. Trotz umfangreicher Bemühungen gelang es nicht, die Quelle dieses Ausbruchs zu identifizieren.

Am 01.03.2020 war ein Skiurlauber aus Ischgl/Tirol nach Island zurückgekehrt und an COVID-19 erkrankt [20]. In der Folge wurde bei 15 Rückkehrern aus Ischgl COVID-19 diagnostiziert, und Island erklärte Ischgl am 05.03.2020 zum Risikogebiet. Nach Recherchen des Bayerischen Rundfunks haben sich auch mindestens 341 Deutsche in Ischgl angesteckt [1]. Laut einer am 25.06.2020 von der Universität Innsbruck veröffentlichten Untersuchung hatten 42,4 % der Bewohner von Ischgl Antikörper gegen SARS-CoV‑2 [44].

Aufgrund der Zunahme der Fallzahlen auch außerhalb Chinas erklärte die WHO am 11. März 2020 den Ausbruch einer Pandemie. Bis dahin wurden bereits 118.000 Fälle aus 114 Ländern gemeldet [49].

Die Weltbevölkerung war praktisch naiv gegenüber diesem neuen Erreger, und eine wirksame Impfung steht bisher (Juli 2020) nicht zur Verfügung. Daher wurde zunächst davon ausgegangen, dass die Epidemie erst nach Erreichen einer Herdenimmunität, also nach Infektion von 60–70 % der Bevölkerung, zum Stillstand kommt. Die Situation in Wuhan legte eine andere Einschätzung nahe: Dort wurde nach 3 Monaten ein erstes Plateau der Erkrankungszahlen, zuletzt unter Lockdown-Bedingungen, bei einer Infektionsrate von ca. 0,7 % der Bevölkerung erreicht. Auf Deutschland übertragen bedeutete dies etwa 560.000 Infektionen in 3 Monaten [33].

Auch wenn die Zahl der bis zum 31. Juli 2020 gemeldeten 209.653 COVID-19-Fälle in etwa den an das Robert Koch-Institut (RKI) gemeldeten Grippefällen in Deutschland gleicht (bis ca. 275.000 Fälle pro Jahr), so hat die Infektion mit SARS-CoV‑2 mit ca. 4,4 % eine höhere Sterblichkeit als die saisonale Influenza [36]. In manchen Regionen lag die Sterblichkeit an COVID-19 über 10 %. Möglicherweise spielten hier eine kapazitative Überforderung des Gesundheitssystems sowie die Tatsache eine Rolle, dass man erst mit der Zeit die pathomechanistischen Tücken dieses neuen Virus erkannte. Die Infektion kann eine Endotheliitis und eine Hyperkoagulabilität hervorrufen und so nicht nur bei vorbestehenden Grunderkrankungen, sondern auch bei gesunden Menschen Thromboembolien und Organversagen auslösen [13, 24, 30, 31, 45].

Zur Beurteilung der Situation wurde zunächst die Verdopplungszeit in Tagen angegeben, dann der R0-Wert (Basis-Reproduktionszahl), der die Anzahl der Neuinfektionen angibt, die von einem Infizierten ausgehen. Bei der Abnahme der Zahl der Neuinfektionen verlieren diese beiden Werte durch eine große Streuung an Vorhersagekraft, sodass man schließlich die 7‑Tages-Inzidenz pro 100.000 Einwohner zugrunde legte. Damit konnten auch regionale Unterschiede und Cluster berücksichtigt werden.

In Deutschland wurden wegen der ansteigenden Fallzahlen von COVID-19 ab dem 16.03.2020 die Schulen und Tagesstätten geschlossen. Am 23.03.2020 erfolgte ein Kontaktverbot, und zahlreiche Geschäfte und öffentliche Einrichtungen wurden geschlossen. In der Folge nahm die Zahl der täglichen Neuinfektionen deutlich ab. Bundesweit lag sie am Höhepunkt der Pandemie am 27.03.2020 bei 6294 Fällen pro Tag, fiel dann in der ersten Juniwoche auf 214 bis 507 Fälle täglich und stieg bis Ende Juli 2020 wieder auf 305 bis 1012 Fälle pro Tag an.

Insgesamt wurden bis zum 31. Juli 2020 in Deutschland 209.653 Infektionsfälle und 9148 Todesfälle gemeldet. Die am stärksten betroffenen Bundesländer sind Bayern (51.068 Fälle), Nordrhein-Westfalen (49.102 Fälle) und Baden-Württemberg (37.272 Fälle). Die geringsten Fallzahlen wurden bisher in Mecklenburg-Vorpommern (868 Fälle), Bremen (1781 Fälle) und Sachsen-Anhalt (2022 Fälle) registriert [35].

Mit Stand vom 31.07.2020 wurden weltweit ca. 17,6 Mio. SARS-CoV-2-Infizierte und ca. 680.000 Todesfälle gemeldet [21]. Die am stärksten betroffenen Länder sind USA, Brasilien und Indien, wobei aus vielen Ländern der Dritten Welt keine verlässlichen Zahlen gemeldet werden können. Von Februar bis Mai 2020 kam es weltweit zu einer ersten Welle, die durch geeignete „Lockdown“-Maßnahmen sowie Hygiene- und Abstandsregeln (Social Distancing) und Maskenpflicht eingedämmt werden konnte.

Ab Juni 2020 kam es in vielen europäischen und außereuropäischen Ländern wieder zur Zunahme der Neuinfektionen [21]; vor allem dort, wo Maßnahmen, die zur Verhinderung der Übertragung des Erregers erlassen wurden, zu früh aufgehoben, oder Sicherheitsregeln wie Mindestabstand und Maskenpflicht nicht konsequent eingehalten wurden. Beispiele sind der Wiederanstieg der Fallzahlen in Israel, Iran, Katalonien/Spanien sowie mehreren US-Bundesstaaten [51–57]. Aus den gleichen Gründen hatte es bereits bei der sog. Spanischen Grippe 1918/1919 in den USA an zahlreichen Orten eine zweite Welle gegeben [5].

Eine solche zweite Welle wird oft durch bestimmte Situationen begünstigt, wie z. B. im Umfeld von Reisenden aus Hochpandemie-Gebieten, durch familiäre Kontakte, Familienfeiern, Begräbnisse, Gottesdienste, in Schulen, bei unzulänglichen Arbeitsbedingungen und überall dort, wo enge Kontakte unzureichend vermieden werden [27].

## Übertragung

Die Übertragung von SARS-CoV‑2 erfolgt im Wesentlichen durch Tröpfcheninfektion bei engem und direktem Kontakt [8].

In experimentell erzeugten Aerosolen zeigte infektiöses SARS-CoV‑2 eine Halbwertszeit von 1,1 h [10], und selbst 90 min nach Aerosol-Erzeugung war noch vermehrungsfähiges SARS-CoV‑2 nachweisbar [40]. In der Literatur gibt es Hinweise für die Übertragung mittels Aerosolen, allerdings scheint dies nicht der Hauptübertragungsweg zu sein [15].

In einer umfangreichen Metaanalyse, die Erkrankungen durch die Coronaviren SARS-CoV‑1, MERS und SARS-CoV‑2 berücksichtigt, konnten Chu et al. [9] zeigen, dass Masken für den Träger die Gefahr, sich bei SARS-CoV-2-infizierten Personen anzustecken, von 17,4 auf 3,1 % vermindert. Dabei war die Schutzwirkung für Masken vom Typ N‑95 (entspricht etwa FFP2) höher, als bei anderen Masken. Ferner reduzierte sich rechnerisch das Ansteckungsrisiko von 12,8 % bei Abständen unter einem Meter auf 2,6 % bei Abständen über einem Meter. Gleichzeitig ergab sich eine Halbierung des Risikos pro Meter zusätzlichen Abstands. In einer anderen Studie konnte gezeigt werden, dass die Filterwirkung von selbstgenähten dreischichtigen Baumwollmasken beim Ausatmen in etwa der Filterwirkung von chirurgischen Masken entspricht [17].

Für die gegenwärtige COVID-19-Pandemie ergeben Modellrechnungen aus den USA, dass schon eine 80 %ige Einhaltung der Maskenpflicht in den Bundesstaaten Washington und New York die Zweimonats-Todesrate um 17–45 % reduzieren könnte. Die Wirkung des Maskentragens ist, ähnlich wie bei der Grippepandemie von 1918, auch bei COVID-19 am größten, wenn bereits damit begonnen wird, bevor die Übertragungsraten hochschnellen [11].

Gelegentlich geben sog. *Super-Spreader* die Infektion an wesentlich mehr Personen weiter, als es die Basis-Reproduktionszahl R0 angibt [42]. Dabei scheint nicht nur die individuelle Virusmenge, sondern auch das individuelle Sozialverhalten bei der Übertragung von SARS-CoV‑2 eine Rolle zu spielen [15].

Eine Studie aus Guangzhou in China untersuchte den möglichen Infektionszeitraum, bezogen auf den Symptombeginn des Infizierenden [16]. Die Infektiosität der mit SARS-CoV‑2 infizierten Personen beginnt demnach wahrscheinlich 2,3 Tage vor Symptombeginn (95 % Konfidenzintervall [KI] 0,8–3,0 Tage) und ist etwa 0,7 Tage nach Symptombeginn (95 % KI 0,2 vor bis 2,0 Tage nach Symptombeginn) am höchsten. Gleichzeitig nimmt die mittels Polymerase-Kettenreaktion (PCR) gemessene Virusmenge ab Symptombeginn stetig ab. Die durchschnittliche Infektiositätsdauer beträgt insgesamt 8–9 Tage [37].

Auf Oberflächen können Coronaviren bis zu 9 Tage lang nachweisbar bleiben [22], allerdings gibt es keine klinischen Daten, die die Übertragung von SARS-CoV‑2 durch Fremdoberflächen bestätigen.

## Klinik

Es wird vermutet, dass zwischen 56 und 86 % der mit SARS-CoV‑2 infizierten Personen Symptome entwickeln [37]. Allerdings wird auch von einer Kreuzfahrt berichtet, bei der nur 24 (17 %) von 128 infizierten Teilnehmern Symptome zeigten [19].

Nach einer chinesischen Studie klagen Patienten mit nachgewiesener SARS-CoV-2-Infektion u. a. über Fieber (92 %), trockenen Husten (53 %), Auswurf (31 %), Abgeschlagenheit (28 %), Myalgien (22 %), Atemnot (14 %) und Durchfall (6 %) [12]. Weiterhin werden Halskratzen, Schnupfen, Atemnot, Störungen des Geruchs- und/oder Geschmackssinns, Kopf- und Gliederschmerzen, gastrointestinale Beschwerden, Konjunktivitis, arterielle oder venöse Thromboembolien, Hautausschlag, Lymphknotenschwellung sowie Apathie und Somnolenz beschrieben.

Eine italienische Studie wies pneumonische Infiltrate mittels Computertomographie (CT) bei 147 von 162 Patienten (91 %) mit PCR-positiver SARS-CoV-2-Infektion und bei 24 von 152 Patienten mit klinisch vermuteter SARS-CoV-2-Infektion (PCR-negativ) nach [6].

In einer chinesischen Studie mit 72.314 COVID-19-Fällen wurden 81 % als mild, 14 % als schwer (Atemnot, verminderte Sauerstoffsättigung, Tachypnöe) und 5 % als kritisch (akutes Lungenversagen [ARDS], Schock, Organversagen) eingestuft [58]. Von den 2087 als kritisch eingestuften Patienten verstarben 1023. Die Sterblichkeit stieg mit dem Alter (8 % der 70-bis 79-Jährigen und 14,8 % der über 80-jährigen).

Adipositas (Body-Mass-Index [BMI] über 30) und Begleiterkrankungen wie koronare Herzerkrankung, Lungenerkrankungen einschließlich chronisch-obstruktive Lungenerkrankung (COPD), Lebererkrankungen, Malignome sowie Immunschwächen erhöhen ebenfalls das Risiko, an COVID-19 zu versterben [50].

## Diagnostik

COVID-19 wird mittels Rachen- oder Nasenabstrich diagnostiziert, der in der PCR auf das Vorhandensein von Ribonukleinsäure (RNA) des Erregers untersucht wird [37]. Bei symptomatischen Patienten mit positivem PCR-Testergebnis ist die Wahrscheinlichkeit ca. 99 %, dass eine Infektion vorliegt. Bei symptomatischen Patienten kann mit nur einem einzigen negativen Testergebnis eine COVID-19-Infektion nicht sicher ausgeschlossen werden; die Wahrscheinlichkeit eines falsch-negativen Ergebnisses liegt in Studien bei ca. 2–29 % [2, 48].

In der Literatur sind Fälle bekannt, bei denen die PCR im Rachenabstrich zunächst negativ war und die Diagnose anhand typischer pulmonaler Veränderungen in der Thorax-CT gestellt wurde [28].

Eine abgelaufene Infektion mit SARS-CoV‑2 kann mit einem Antikörpernachweis im Serum festgestellt werden. Während die herkömmlichen Elisa-Tests schon eine relativ hohe Sensitivität aufweisen (wenig falsch-negative Befunde), erscheint die Spezifität (falsch-positive Befunde) verbesserungswürdig. Daher sollen mittels ELISA erhobene positive Antikörperbefunde mit einem Bestätigungstest, z. B. Immunfluoreszenz, überprüft werden [34]. Es gibt auch Hinweise, dass die Antikörperbefunde bei einem Teil der Patienten innerhalb einiger Monate nach der Infektion wieder negativ werden [29].

## Therapeutische Interventionen

Eine randomisierte Multicenterstudie zeigte, dass die Sterblichkeit bei schwer kranken beatmeten Patienten durch Dexamethason (6 mg täglich bis zu 10 Tage) von 41 auf 29 % gesenkt wurde [32]. Dies traf nicht für Patienten zu, die weder mechanische Beatmung noch Sauerstoffgabe benötigten. Eine britische Netzwerk-Metaanalyse zeigte zusammenfassend, dass bei COVID-19-assoziiertem ARDS von verschiedenen unabhängigen Interventionen (Steroide, Beatmung, Hydroxychloroquin, Remdesivir) nur Steroide und Beatmung einen Nutzen für das Überleben brachten [39]. Durch frühzeitige Diagnostik und Therapie von Komplikationen, wie z. B. Thrombosen und Lungenembolien, kann die Sterblichkeit weiter reduziert werden [13, 24].

Remdesivir ist eine „Prodrug“ eines Adenosin-Analogons, das ursprünglich für die Therapie der Hepatitis C entwickelt wurde [62]. Eine doppelblinde randomisierte placebokontrollierte Multicenterstudie ergab eine Reduktion der Dauer des Krankenhausaufenthalts bei COVID-19, während die Reduktion der Todesrate von 11,9 auf 7,1 % nicht signifikant war [3]. Eine ähnliche chinesische Studie [47] und auch die bereits zitierte Netzwerk-Analyse [39] haben keinen signifikanten klinischen Nutzen für Remdesivir ergeben. Aus diesen Gründen wird in der Literatur derzeit nur eine schwache Empfehlung für den Einsatz von Remdesivir ausgesprochen [38].

Weitere antivirale Medikamente, die einen In-vitro-Effekt gegen das SARS-CoV‑2 zeigten, und auch Immunmodulatoren sind derzeit in klinischer Erprobung; ihre klinische Wirksamkeit lässt sich noch nicht abschätzen.

Initial wurden große Hoffnungen auf eine mögliche Wirkung von Chloroquin und das besser verträgliche Hydroxychloroquin gegen SARS-CoV‑2 gesetzt. Dies wurde jedoch in größeren randomisierten Studien und Metaanalysen nicht bestätigt [7, 39]. Vielmehr zeigte eine brasilianische Studie eine dosisabhängige Gefährdung der Patienten durch Hydroxychloroquin [41].

## Impfung

Von mehr als 170 laufenden Impfstoff-Projekten sind im Juli 2020 ca. 30 Impfstoff-Kandidaten bereit, in klinische Phase-1- bis Phase-3-Studien einzutreten [46]. Attenuierte Viren, gentechnisch hergestellte Proteine oder Proteindomänen (z. B. des S1-Proteins), DNA- und RNA-Impfstoffe sowie Impfstoffe mit Trägerviren, wie beispielsweise Masern- oder Adenoviren, werden derzeit auf Sicherheit, Verträglichkeit und Wirksamkeit (Bildung neutralisierender Antikörper) untersucht. Vereinzelt gibt es Berichte, dass Antikörper nach COVID-19-Infektionen zeitnah wieder verschwinden [29]. Hier gilt es, Impfstoffe zu finden, die mithilfe effizienter Adjuvanzien eine lang anhaltende humorale und zelluläre Immunität hervorrufen. Mit ersten zugelassenen Impfstoffen kann voraussichtlich Ende 2020 bzw. Anfang 2021 gerechnet werden.

## Wirtsantwort und Pathologie

Angiotensin Converting Enzyme 2 (ACE2), ein Regulator des Renin-Angiotensin Systems, ist membrangebunden und wirkt als Rezeptor für das Spike-Protein des SARS-CoV‑2 [63]. Zahlreiche Zellarten exprimieren ACE2, darunter Epithelzellen des Nasopharynx, der Lungen (Alveolarzellen), des Gastrointestinaltrakts und der Haut. ACE2 kommt u. a. auch in Lymphknoten, Nieren, Herz, Gehirn, Knochenmark und Milz vor [14]. Es ist auch in den arteriellen und venösen Endothelzellen sowie in den glatten Muskelzellen der Arterien der genannten Organe nachweisbar.

Schwere Verläufe von COVID-19 sind in der Regel von einer starken Aktivierung der Immunabwehr begleitet [61]. Beobachtet werden u. a. eine Lymphozytopenie, eine Vermehrung der Neutrophilen sowie erhöhte Serumspiegel von IL-1β, IL‑2, IL‑4, IL‑6, IL-10, TNF‑α, und Interferon‑γ. Diese Zytokinantwort ist mit einer Zellschädigung assoziiert, die sich wiederum in erhöhten Serumspiegeln u. a. von Laktatdehydrogenase (LDH), Herz- und Leberenzymen sowie einer Aktivierung von Gerinnung und Fibrinolyse mit stark erhöhten Plasmaspiegeln der D‑Dimere widerspiegelt. Bei ARDS durch COVID-19 ist ein Alveolarschaden mit Desquamation von Pneumozyten, hyalinen Membranen und lymphomonozytären Infiltraten beschrieben [60]. Eine andere Arbeit berichtet von lymphozytärer Endotheliitis und Apoptose der Endothelzellen in Lunge, Nieren und Dünndarm [45].

Der Endothelschaden dürfte zu der Hyperkoagulabilität sowie zu Thrombosen und Embolien beitragen, die bei schweren Verläufen von COVID-19 beobachtet werden [13, 24, 30, 31]. Ebenso lassen sich dadurch Komplikationen wie das Auftreten Kawasaki-ähnlicher Erkrankungsbilder bei Kindern und Jugendlichen [18, 43] und Vaskulitiden verschiedener Organe erklären [45]. Auch im Gehirn zeigen MRT-Untersuchungen enzephalitische, hämorrhagische, thromboembolische, ischämische und leukenzephalopathische Läsionen [23, 26]. Somit ergeben sich bei COVID-19 pathophysiologische Veränderungen, die sich nicht nur in der Lunge, sondern in einer Reihe anderer Organe in der radiologischen Bildgebung spezifisch darstellen lassen.

## Fazit für die Praxis

- SARS-CoV‑2 wird über Tröpfchen und wahrscheinlich auch Aerosole übertragen.
- Die Infektiosität beginnt 2–3 Tage vor Symptombeginn; auch asymptomatisch Infizierte sind infektiös.
- Die Erkrankung betrifft die oberen Atemwege und die Lungen (Pneumonie, akutes Lungenversagen [ARDS]), ferner Herz, Leber, Magen-Darm-Trakt und andere Organe.
- Gefährdet sind insbesondere ältere Menschen sowie Personen mit Vorerkrankungen.
- Eine effektive antivirale Therapie ist bisher nicht verfügbar.
